# Can atypical antipsychotics alleviate Deficits in psychosocial impairments in patients with a diagnosis of Borderline Personality? A systematic review and meta-analysis

**DOI:** 10.1016/j.psycom.2024.100187

**Published:** 2024-09

**Authors:** Katie Griffiths, Nadezhda Velichkova, Lisa Quadt, Jimena Berni

**Affiliations:** Department of Clinical Neuroscience, Brighton and Sussex Medical School, University of Sussex, UK

## Abstract

Patients with a diagnosis of Borderline Personality Disorder (BPD) often experience difficulties in psychosocial functioning, which reduces the ability of individuals to engage socially. This review seeks to determine whether atypical antipsychotics (AAPs) are more effective than placebo at alleviating these difficulties in adults with a diagnosis of BPD.

We identified six Randomized Control Trials, conducted between 1994 and 2024, with 1012 patients that were treated with either: Olanzapine, Quetiapine, Ziprasidone or Aripiprazole. Using a meta-analysis, we found evidence that atypical antipsychotics induce a small improvement treating psychosocial functioning in patients with a diagnosis of border line personality. In particular, AAPs improved General Assessment of Functioning (GAF) more than placebo. Combining GAFs P-values from several studies indicated this effect was significant. AAPs were also superior to placebo at improving quality of interpersonal relationships, occupational functioning and family life. There was a positive improvement tendency in social life and leisure activities. AAPs also induced known secondary effects like weight gain and sedation as previously described.

AAPs were beneficial for improving general functioning and its subcomponents. However, the magnitude of the benefit above that of placebo was small and its clinical meaningfulness is thus debatable. More randomised-controlled trials are required.

## Highlights

• Atypical antipsychotics alleviate Deficits in psychosocial impairments in patients with a diagnosis of border line personality.
• General Assessment of Functioning is improved compared to placebo.
• Quality of interpersonal relationships, occupational functioning and family life also show a small improvement.

## 1 Background

Borderline personality disorder (BPD; also referred to as Emotionally Unstable Personality Disorder: EUPD) is a condition characterized by persistent and debilitating mood regulation difficulties, negative self-image, and interpersonal dysfunction and conflict (Black and Grant, 2014). It has a prevalence of 1.6% within the general population and is associated with more disability-adjusted life years relative to other mental health disorders (Chapman et al., 2024). Difficulty in overall functioning is a well-recognised and often pervasive consequence of the condition, in which individuals' abilities to engage socially are diminished. This compounds individuals' distress and BPD's societal burden (Fariba et al., 2023). Although BPD symptom severity tends to diminish over adult life, functional difficulties are more resistant to this natural decline, persisting even after an individual has met the criteria for remission (File et al., 2017; Zanarini et al., 2005). This makes functioning a valuable therapeutic target and increasingly an outcome of interest in clinical trials (Zanarini and Frankenburg, 2015). Nevertheless, no existing systematic review has analysed the effects of atypical antipsychotics (AAPs), a class of commonly prescribed drugs in BPD, on psychosocial function.

Over 90% of patients are prescribed medications for BPD, with up to 60% of these being antipsychotics (Hancock-Johnson et al., 2017). AAPs are a group of psychotropic medications acting to varying degrees on dopamine, serotonin, histamine, and noradrenergic receptors; each AAP differs with respect to its exact pattern of receptor-binding affinities (Belli et al., 2012). Through interactions with these receptors, AAPs alter the activity of the corresponding neurotransmitter and this may modulate affect (Fariba et al., 2023). Since dysregulation of affect and emotional processing appears to underpin important aspects of BPD's pathophysiology, AAPs may thereby work by better regulating these processes (Fariba et al., 2023). Despite their widespread use, no consensus has been reached on the efficacy and role of AAPs in the treatment of BPD symptomatology, and The British National Institute for Health and Care Excellence (NICE) guidelines consequentially do not explicitly endorse their use (NICE, 2018; Schrift, 2023). Certain symptom domains appear responsive to AAPs, but it is disputed whether the benefit is clinically meaningful, and it remains unknown whether benefits extend to psychosocial functioning (Hancock-Johnson et al., 2017; Ripoll et al., 2011). Additionally, their side effects can be burdensome and include weight gain, asthenia, sexual dysfunction, and sedation, consequently compromising metabolic health, self-image, and potentially psychosocial functioning (Fariba et al., 2023; NICE, 2018). Any benefits must therefore be considered in proportion with these risks.

### 1.1 Aims

This systematic review aimed to summarise and interpret the impact of AAPs on psychosocial functioning in patients with a diagnosis of BPD and map their risk-to-benefit profile.

## 2 Methods

### 2.1 Search strategy

This systematic review was conducted in accordance with Cochrane Collaboration, and Preferred Reporting Items for Systematic Reviews and Meta-analyses (PRISMA) guidelines (Page et al., 2021). The search was conducted by KG and revised by JB. The following electronic databases for published and unpublished studies available in the English language between January 1994 and December 2021 were searched: PubMed, EMBASE, and Cochrane Central Register of Controlled Trials and Clinical Trial Registries. Studies conducted before 1994 were excluded as these pre-dated the release of DSM-IV, in which BPD was more explicitly defined (Silk, 2002). The following terms were used to search in titles, abstracts, and as medical subject heading terms: “borderline personality disorder”, “emotionally unstable personality disorder”, and “atypical antipsychotic”. Additional searches were performed with the names of individual AAPs. The full search terms are provided in the Appendix. References of included studies and relevant reviews were snowball searched for further studies. Eligible studies included adults of any gender aged over 18 years and diagnosed with BPD/EUPD according to DSM-IV, DSM-V, or ICD-10 criteria without co-occurring psychiatric diagnoses. To improve reliability only randomised placebo-controlled trials were included. Studies were excluded if they did not report at least one domain of psychosocial functioning.

An updated search was conducted on the 1st of March on pubmed using the “*name of each AAP* borderline personality trial”. One paper was found for Clozapine, but discarded after assessment since the data did not reach power.

The clinical trial registration number was used to identify all published reports for comprehensive extraction of data. Titles and abstracts of articles were screened by one author, KG, to produce a shortlist of potential articles. The full texts were then reviewed for eligibility as per the a priori criteria. Authors of studies with missing data were contacted for provision of information.

### 2.2 Outcome measures

Functioning can be assessed globally or divided into subcomponents, including occupational functioning and social functioning. The primary outcome was the longitudinal change in global functioning, as measured by the Global Assessment of Functioning (GAF) scale. Secondary outcomes were social and occupational functioning, as well as dropout rates and reported side effects. These subcomponents of functioning were assessed using the Sheehan Disability Scale (SDS), the Symptom Checklist-90 Revised (SCL-90-R), the Zanarini Rating Scale for BPD (ZAN-BPD), Borderline Personality Disorder Severity Index (BPDSI), and the Clinical Global Impression BPD scale (CGI-BPD).

GAF is a clinician-reported assessment of how much a person's day-to-day psychological, social, and occupational functioning is affected by their symptoms. It is measured on a scale from 0 to 100, with a higher score representing superior functioning (Aas, 2010). The SDS is a self-reported tool (Sheehan, 1983). Patients use a 10-point Visual Analogue Scale (VAS) to assess how much their symptoms disrupt their functioning within the following three items: Work or School Life, Family Life or Home Responsibilities, and Social Life or Leisure Activities. A score of 5 or more in any individual item is considered clinically significant (Sheehan, 1983). SCL-90-R is a self-administered questionnaire evaluating 90 psychological symptoms. It asks individuals to rate each symptom's severity from 1 to 5, with 1 indicating no symptoms, and 5 indicating severe symptoms (Vaurio, 2011). These items are then combined to evaluate nine symptomatic dimensions, one of which is Interpersonal Sensitivity which was included in this review. ZAN-BPD is a clinician-administered instrument following a patient interview (Zanarini et al., 2003). It uses a five-point rating scale for each of the nine criteria for BPD described in DSM-IV/5. The Instability in Interpersonal Relationships domain was included in this review. BPDSI is a 70-item semi-structured clinical interview assessing the frequency and severity of manifestations of BPD symptoms, including quality of interpersonal relationships (Arntz et al., 2003). CGI-BPD is a clinician-reported scoring system which contains 10 items scoring nine psychopathological domains of BPD plus a global score (Perez et al., 2007). The Unstable Relations item was included in this review.

### 2.3 Data collection

Data extraction was performed by KG and revised by JB, using piloted forms developed according to PRISMA guidelines, which included population, intervention, comparison, outcomes, and setting (PICOS) data items (Page et al., 2021).

### 2.4 Data analysis

When sufficient data was available, meta-analyses were conducted using Rev. Manager 5 software (RevMan, 2008). Standardised mean difference (SMD) was adopted as a measure of effect size, computed as the difference between the mean of the patient and control groups (group mean scores at endpoint, or mean baseline-to-endpoint changes), divided by the standard deviation, and weighted for sample size. SMD was used to avoid bias derived from small sample sizes, and because different papers used different outcome scores (Higgins et al., 2022). Given the heterogeneity in the measures of psychosocial function, the generic inverse variance model and random effects were used to estimate the pooled SMDs of the included studies. Data from multi-arm trials were merged using formulae for combining groups and online calculators in Rev. Manager. When change scores were not reported, these were calculated by subtracting endpoint value from baseline. When baseline measures were absent, only endpoint data was used. This relied on the assumption that baseline values did not significantly differ between groups and sensitivity analysis was subsequently performed with these studies excluded (Higgins et al., 2022). The heterogeneity between studies was investigated using Chi-square test of homogeneity and I_2_-statistic. Values of 50% heterogeneity and higher were considered non-negligible, and explanations in the included studies were searched for (Higgins et al., 2022).

When insufficient data was present for meta-analysis, data was analysed using a direction of effect analysis plus combination of P-values as per current Cochrane guidelines (McKenzie and Brennan, 2019). Direction of effect analysis identifies whether baseline-to-endpoint changes favour AAP or placebo condition. Importantly, it does not consider whether any between-group differences reach statistical significance, and simply reports the trends. The sign test is a nonparametric test used to interpret the direction of effect analysis. To perform the test, the number of positive and negative effect direction arrows for each outcome domain were counted and GraphPad Prism vs9.0 was used to calculate a one-tailed P-value for each outcome (Ruxton and Neuhuser, 2010). The P-value represents the probability of observing the same pattern of positive and negative results if the null hypothesis of an equal number of positive and negative results is true. Combination of P-values using Fisher's method was performed according to current Cochrane guidelines (Appendix) (McKenzie and Brennan, 2019). A P-value of less than 0.05 (two-tailed) was considered statistically significant for all calculations. All data is presented as mean (± standard deviation), unless otherwise stated.

### 2.5 Assessment of risk of bias

The Revised Cochrane risk-of-bias tool for randomised trials (RoB 2) was used to evaluate risk-of-bias (Sterne, 2019). Bias was assessed at both the study and outcome level, and included assessments of random sequence generation, allocation concealment, and incomplete or selective data reporting.

## 3 Results

### 3.1 Study selection

**Fig. 1:**
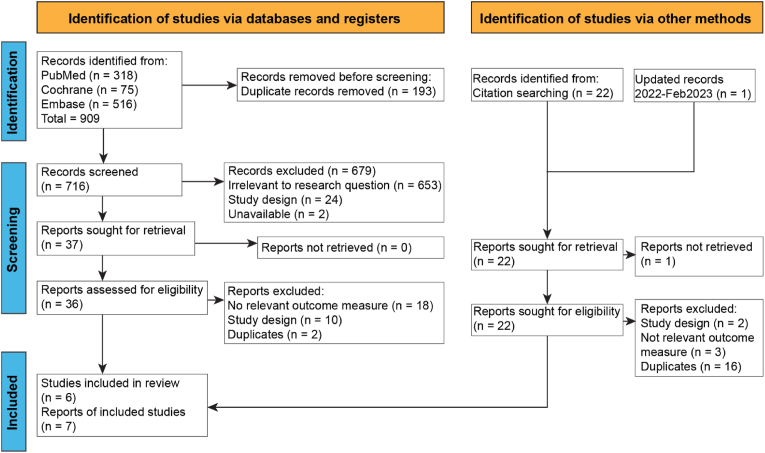
– Flow diagram of study selection.

The search strategy retrieved a total of 908 publications from all sources. 193 duplicates were removed. Abstracts and titles of the remaining 715 reports were screened. 653 were excluded as they were not relevant to the study question, 24 due to study design, and 2 as they were unavailable. 36 full texts were then reviewed which generated 7 reports of 6 Randomized Control Trials (RCTs) conducted between 2004 and 2017, involving a total of 1012 subjects (Fig. 1).

### 3.2 Study characteristics

Study characteristics and findings are presented in Table 1. All studies were conducted in the outpatient setting and included both females and males (72% = females). In total, 595 patients received AAP treatment, and 417 received placebo. Assessment tools included the SDS (n = 3), Zanarini-BPD (n = 3), SCL-90-R (n = 3), GAF (n = 3), BPDSI (n = 1), and CGI-BPD (n = 1). Trial duration ranged from 8 to 72 weeks (mean: 20.7 ± 23.4 weeks). Six reports described short-term findings (duration of 8 to 12-weeks), and one report was an 18-month extension of an existing 8-week long study. Both reports were included in systematic review, and the 8-week study only was included in further analysis.

**Table 1:** Summary of study characteristics and results. Data is presented as mean ± standard deviation, unless otherwise stated. (AAP = atypical antipsychotic, RCT = randomised-controlled trial, N = number of participants, SE = standard error, AAP = atypical antipsychotic, F = Female, NS = non-significant. *Chlorpromazine dose equivalent, source: Psychiatric Pharmacy Essentials: Antipsycotic Dose Equivalents*, n.d.)*

Study	Design, allocation ratio, duration of intervention	Sample size, Gender (%)	AAP (dosage per day), Chlorpromazine equivalent per day*	Summary of findings		
				Outcome measure	Result	Qualitative description
Pascual et al. (2008)	Double-blind RCT, Ratio = 1:1, 12 weeks	N = 60, F = 82%	Ziprasidone (40–200 mg), 67–333 mg	CGI-BPD; Unstable relations	End-of-study observation. AAP: 4.37 ± 1.1, Placebo: 4.50 ± 1.0, (P = 0.2074)	NS between-group differences at endpoint.
Black et al. (2014)	Triple-blind RCT, Ratio = 1:1:1, 8 weeks	N = 95, F = 54%	Quetiapine (150 mg or 300 mg), 200 mg or 400 mg	GAF	Mean weekly change (±SE). AAP: 1.05 ± 0.20, Placebo: 0.62 ± 0.19	NS differences between groups' mean weekly change.
				SDS; Total score	Mean weekly change (±SE). AAP: −0.98 ± 0.23, Placebo: −0.58 ± 0.18.	NS differences between groups' mean weekly change.
				SDS; Work/School score	Mean weekly change (±SE). AAP: −0.28 ± 0.08. Placebo: −0.10 ± 0.07.	Both Quetiapine dose groups had significantly greater mean weekly reduction than placebo.
				SDS; Family Life score	Mean weekly change (±SE). AAP: −0.38 (±0.11). Placebo: −0.25 (±0.07).	NS differences between groups' mean weekly change.
				SDS; Social life score	Mean weekly change (±SE). AAP: −0.32 ± 0.10. Placebo: −0.22 ± 0.08.	NS differences between groups' mean weekly change.
				ZAN-BPD; Disturbed relationships	Weekly change (±SE). AAP: −0.30 (±0.06), placebo: −0.18 (±0.05).	Both Quetiapine dose groups had significantly greater weekly reductions than placebo.
Nickel et al. (2006)	Double-blind RCT, Ratio = 1:1, 8 weeks	N = 52 F = 83%	Aripiprazole (15 mg), 200 mg	SCL-90-R; Insecurity in social contacts	End-of-study observation. AAP: 59.7 (±5.3), Placebo: 64.2 (±6.2).	Aripiprazole resulted in a significantly greater rate of improvement than placebo.
Nickel et al. (2007)	Double-blinded RCT Ratio = 1:1 18 months	N = 52 F = 83%	Aripiprazole (15 mg), 200 mg	SCL-90-R; Insecurity in social contacts	End-of-study observation. AAP: 57.2 (±7.3), Placebo: 67.0 (±9.0) (P < 0.01).	Aripiprazole resulted in a significantly greater improvement than placebo.
Schulz et al. (2008)	Double-blind RCT, Ratio = 1:1, 12 weeks	N = 314 F = 71%	Olanzapine (2.5–20 mg), 50–400 mg	SDS; Family life score	Mean endpoint to baseline change (±SE). AAP: −2.05 (±0.24), Placebo: −1.39 (±0.23).	Olanzapine resulted in significantly greater mean improvements than placebo.
				SDS; Work/School score	Measured but not reported.	–
				SDS; Social life score	Measured but not reported.	–
				SCL-90-R; Insecurity in social contacts	Data not obtainable; direction of effect in favour of AAP.	NS differences between groups' longitudinal change.
				GAF	Measured but not reported.	–
Zanarini et al. (2011)	Double-blind RCT, Ratio = 1:1:1, 12 weeks	N = 439 F = 74%	Olanzapine (2.5 mg, or 5–10 mg), 50 mg, or 100–200 mg)	GAF	Mean longitudinal change. AAP: 10.3, Placebo: 7.8, (P = 0.111).	NS differences between groups' longitudinal change.
				SDS; work/school score	Mean longitudinal change. AAP: −2.50, P: −1.80, (P = 0.072).	NS differences between groups' longitudinal change.
				SDS; social life score	Mean longitudinal change. AAP: −2.80, Placebo: −2.20, (P = 0.096).	NS differences between groups' longitudinal change.
				SDS; family life score	Mean longitudinal change. AAP: −2.90, Placebo: −2.10, (P = 0.014).	Significantly greater improvement following Olanzapine treatment.
				ZAN-BPD; Instability in interpersonal relationships	Mean longitudinal change. AAP: −0.95, Placebo: −0.80, (P = 0.353).	NS differences between groups' longitudinal change.
Bogenschutz et al. (2004)	Double-blind RCT, Ratio = 1:1, 12 weeks	N = 40 F = 63%	Olanzapine (mean dose: 6.9 mg ± 3.2, maximum dose 20 mg), 138 ± 64 mg, maximum 400 mg	CGI-BPD; unstable interpersonal relationships	Mean longitudinal change. AAP: −2.13 (±1.75), Placebo: −1.53 (±2.14).	NS differences at any timepoint.
				SCL-90-R; insecurity in social contact	Not obtainable.	NS differences at any timepoint.
				GAF	Not obtainable.	NS between groups differences endpoints. Significant between-group differences at week 8.

### 3.3 Assessment of study quality

**Fig. 2:**
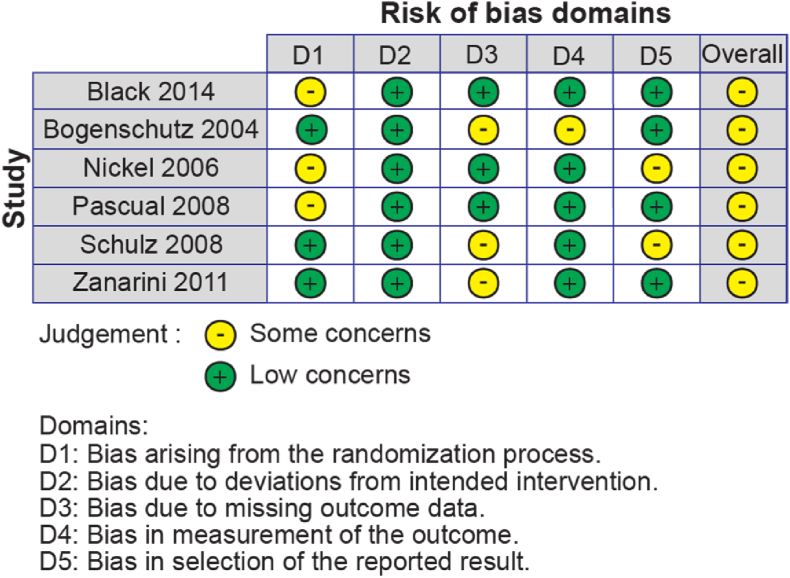
– Summary of the risk of bias of each included study according to the review author's judgement using the criteria and procedure outlined by RoB 2 (Sterne, 2019).

Assessments of study quality according to RoB 2 are provided in Fig. 2. All studies were judged to have some concerns regarding their risk of bias. All six studies were randomised and double-blinded. However, only three studies described the process of sequence generation for randomisation (1 used web software, 2 used randomisation codes). Three studies failed to report data when results were non-significant, which may cause bias due to missing data.

### 3.4 Therapeutic effects

#### 3.4.1 Global functioning

**Fig. 3:**
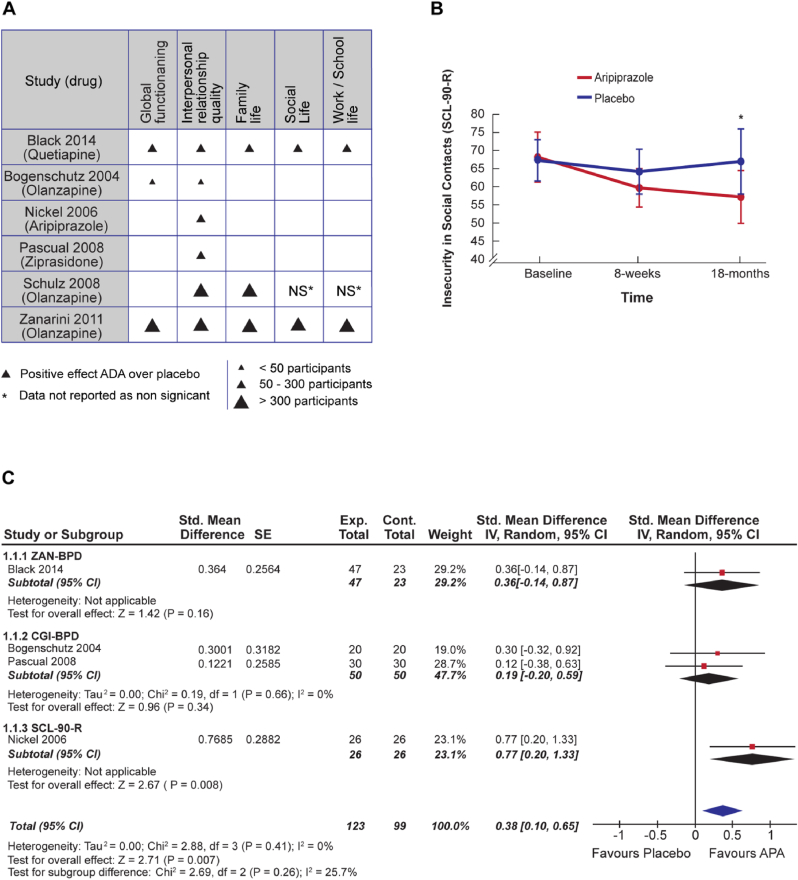
–Summary of the effect of AAP on different Psychosocial aspects. A) Description of the direction of effect for each measure of psychosocial function in all relevant studies. **Direction of effect:** Arrowhead pointing up = positive effect of AAP over placebo. **Sample size:** Large arrow = >300 participants, medium arrow = 50–300 participants, small arrow =<50 participants. NS* means that the actual data were not reported because they were non-significant. When data were not collected the box was left blank. B) Summary of the results of the study which investigated the change in participants' insecurity in social contacts according to the SCL-90-R scale over an 18-month period following Aripiprazole or placebo treatment. Each data point plus error bar represents the mean ± Standard deviation. * = P < 0.01. Data derived from (Nickel et al., 2006, 2007). C) Forest plot presenting the results of the meta-analysis which investigated the change in interpersonal relationship quality following AAP versus placebo treatment at endpoint. For each of the four included studies the standardised mean difference (std. mean difference), associated 95% confidence intervals, and sample size have been listed and displayed. The measures below the forest plot present the amount of heterogeneity present among this sample of studies, the test for the overall effect, and the test for subgroup differences. Note that the length of all studies was not identical. Exp. = experimental; Cont. = control.

Three studies with a total of 544 participants, that reached the end of the trial, reported global functioning using the GAF scale (Black et al., 2014; Bogenschutz and George Nurnberg, 2004; Zanarini et al., 2011). These three studies reported a trend towards increased improvement in GAF following AAP treatment but not placebo, although this never reached statistical significance at endpoint. Despite AAPs out-performing placebo in all trials, the direction of effect analysis provided insufficient evidence of effect due to the low number of included studies (Fig. 3A). The sign test indicated that the probability of observing this same pattern of results was too high to reject the null hypothesis that AAPs were not more effective than placebo (P = 0.13).

Only one study (Bogenschutz and George Nurnberg, 2004) reported statistically significant benefits of AAPs at all timepoints. However, between-group differences were only significant at 8-weeks of trial duration (P = 0.036), and the authors failed to report endpoint values (12-weeks) due to statistical non-significance. In the two studies which adequately reported endpoint data, the mean weekly change in GAF was 0.95 (±0.14) in AAP groups and 0.64 (±0.02) in placebo groups (Fig. 3A).

Combination of P-values for the four studies provided evidence of superiority of AAP treatment at endpoint compared to placebo (X_2_ = 21.74, P < 0.001).

#### 3.4.2 Social functioning

Social functioning was divided into assessments of quality of interpersonal relationships, quality of social life and leisure activities, and quality of family life to reflect the different perspectives provided by each measurement tool.

*Quality of interpersonal relationships -* All studies reported an endpoint measurement of quality of interpersonal relationships. Four reported sufficient data for pooling using meta-analysis, which indicated a small but significant overall effect in favour of AAPs (SMD = 0.38; 95% confidence interval (CI) = [0.1, 0.65]; P = 0.007, Fig. 3C). The Chi-squared test for heterogeneity was not significant (I_2_ = 0%, P = 0.41) indicating study results were sufficiently similar to warrant their combination. Additionally, all six studies reported an effect direction in favour of AAP use, which the sign test indicated was significant (P = 0.02). Combining of P-values for these six studies also implied significant global effect (P < 0.001).

Additionally, Nickel et al. (2007) indicated that with continued AAP prescription, insecurity in social contacts as measured by SCL-90-R continued to reduce over 18-months after an initial 8-week intervention (Fig. 3B) (Nickel et al., 2006). In contrast, the level of insecurity in social contacts returned to baseline in the placebo group despite no change in protocol. The difference between groups was considered significant at endpoint (P < 0.01) but not at initial evaluation (P = 0.61). A significant group x time effect (P < 0.01) using a two-factor repeated measure analysis of variance was reported by Nickel et al. (2007).

*Social life and leisure activities -* Two studies (Black et al., 2014; Zanarini et al., 2011) reported the Social Life item of the SDS. Both reported a direction of effect in favour of AAP versus placebo, however the sign test indicated that the number of studies was too small for this effect to be considered statistically significant (P = 0.25). Although neither study reported statistically significant differences between groups at endpoint, combining of P-values of both studies indicated an overall significant effect (P = 0.01). Regarding the clinical meaningfulness of Black et al. (2014)'s results, neither Quetiapine nor placebo elevated individuals from the severely impaired category of functioning (SDS <5) by endpoint. Zanarini et al. (2011)'s participants' baseline score exceeded five, and therefore could not be interpreted.

*Family Life -* Three studies reported the Family Life item of the SDS (Black et al., 2014; Schulz et al., 2008; Zanarini et al., 2011). Their mean change in score per week was 0.26 (±0.11) in AAP groups and 0.18 (±0.07) in placebo groups. In all three studies, the direction of effect favoured AAP use, although this effect was deemed non-significant owing to limited sample size (P = 0.13). Two studies were pooled using meta-analysis and indicated a small, but significant superiority of AAPs versus placebo (SMD = 0.24; 95% CIs [0.04, 0.44]; P = 0.02) (Black et al., 2014; Schulz et al., 2008). Analysis of heterogeneity indicated non-significant differences in results across these two studies (I_2_ = 0%, P = 0.78). All mean baseline scores exceeded five.

#### 3.4.3 Occupational functioning

Two studies reported the Sheehan Work/School Life score (Black et al., 2014; Zanarini et al., 2011). The mean change in score per week was 0.24 (±0.04) in AAP groups and 0.13 (±0.03) in placebo groups. In both studies, the direction of effect was in favour of AAPs, although the sign test indicated that this effect did not reach statistical significance (P = 0.25). However, combining P-values indicated a significant overall benefit derived from at least one study (P = 0.003). There was heterogeneity in findings as Black et al. (2014) reported a significant benefit of Olanzapine relative to placebo at endpoint (P = 0.05), whilst Zanarini et al. (2011) reported no significant effect of Quetiapine (P = 0.17). Furthermore, Black et al. (2014) demonstrated that Quetiapine facilitated patients' escape from a highly impaired category (SDS <5), whilst placebo did not. Zanarini et al. (2011)'s participants' baseline score exceeded five and could therefore not be interpreted.

#### 3.4.4 Dropout rates and side effects

The most commonly reported side effects were weight gain, sedation, and headaches. Another notable adverse effect was metabolic derangements, including derangements of blood lipids, blood glucose, and blood pressure (Table 2). Additionally, mean dropout rate was 41% (±10%) (Placebo: 34% (±11%), AAP: 47% (±8%)). Pascual et al. (2008) reported that 30% of dropouts were secondary to side effects in the Ziprasidone group, compared to 0% in the placebo group. Similarly, Bogenschutz and George Nurnberg (2004) reported that 20% of patients receiving Olanzapine discontinued due to side effects (10% to sedation, 10% to weight gain). This compared to 0% in the placebo group. Conversely, SCHULZ et al. (2008) reported that an equal percentage of patients (11%) dropped out due to side effects in Olanzapine and placebo groups.

**Table 2:** – Summary of the main side effects reported by all studies. The direction of effect and any summary statistic is presented, if reported by the primary study. *This study relied on spontaneous reporting by participants, all other studies systematically reported side effects. (-= not reported, NS = non-significant).

Study	AAP	Weight gain	Sedation	Headache	Metabolic health
Pascual et al. (2008)*	Ziprasidone	Direction of effect not reported; NS differences between groups.	Increased in AAP group; AAP: 6, Placebo: 0 (P = 0.039),	Increased in placebo group. Placebo: 1, AAP: 0.	–
Black et al. (2014)	Quetiapine	Greater in AAP group; Mean change in weight. Placebo: 0.5 kg (±2.0), AAP: 0.9 kg (±3.8).	Greater in AAP group; AAP: 80.5%, Placebo: 52%.	–	Direction of effect not reported. NS differences in changes in blood lipid or glucose levels.
Nickel et al. (2006)	Aripiprazole	Direction of effect not reported; NS differences between groups.	–	–	–
Nickel et al. (2007)	Aripiprazole	Direction of effect not reported; NS weight changes were observed.	–	Increased in AAP; AAP: 34.6%, Placebo: 30.8%.	–
Schulz et al. (2008)	Olanzapine	Increased in AAP; Mean change, AAP: 2.86 kg (±3.02), placebo: −0.35 kg (±2.68), (P < 0.001).	Increased in AAP; AAP: 11.6%, Placebo: 1.3% (p < 0.001).	Increased in AAP; AAP = 14.8%, Placebo = 1.3% (P = 0.40).	Increased in AAP; Greater increase in fasting total cholesterol (P = 0.003) and Fasting LDL (P = 0.007).
Zanarini et al. (2011)	Olanzapine	Increased in AAP; AAP: 14% Placebo: 0.7% (P < 0.05).	Increased in AAP; AAP = 18.2, Placebo = 6.5%, (P < 0.05).	Increased in Placebo; AAP = 11.0%, Placebo = 14.4%.	–
Bogenschutz et al. (2004)	Olanzapine	Increased in AAP; Mean change, AAP: 3.71 kg (±3.4), Placebo: 0.17 kg (±4.8). P = 0.027.	Increased in AAP.	–	–

**Fig. 4:**
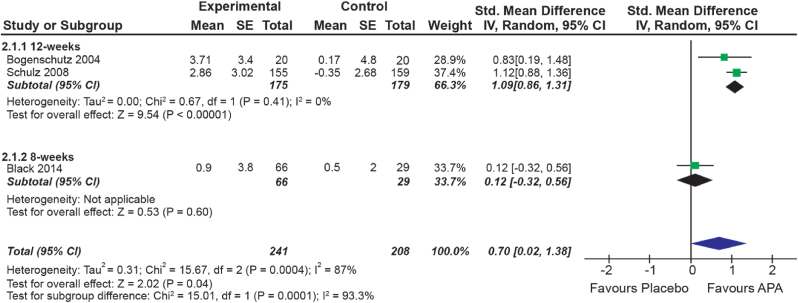
– Forest plot presenting the results of a meta-analysis comparing the mean weight change in patients treated with AAP versus placebo. Subgroup analysis was also performed for trial duration. For each of the 3 included studies the standardised mean difference, their associated 95% confidence intervals, and their sample size has been listed and displayed. The measures below indicate the amount of heterogeneity present among this sample of studies (I_2_), the test for the overall effect, and the test for subgroup differences.

Four trials reported significantly greater weight gain in the AAP group relative to placebo; reporting a mean weekly change in weight by 0.22 kg (±0.08) in AAP groups, versus 0.02 kg (±0.04) in placebo groups. Sufficient data was available for three of these studies to be pooled using meta-analysis which indicated a small, but significant overall effect (SMD = 0.7, 95% CI [0.02,1.38], P = 0.04). The Chi-squared test indicated significant heterogeneity between studies (I_2_ = 87%, P < 0.001); the two 12-week studies reported greater increases in weight than the 8-week study (Fig. 4). Two studies did not report data on weight gain as between-group differences were not statistically significant and could therefore not be included in analysis. Nickel et al., 2006, Nickel et al., 2007 reported no significant difference in weight gain between groups at 8-weeks or 18 months.

Five studies that collected data regarding sedation reported a higher incidence in the AAP group relative to placebo (sign test; P = 0.03). All studies administering Olanzapine, Quetiapine, and Ziprasidone noted an increased incidence of sedation in the active group versus control group (Table 2). No such observations were noted by the study involving Aripiprazole, although this may be due to non-reporting of data (Nickel et al., 2006, 2007). Black et al. (2014) reported that experiencing sedation increased risk of discontinuation, and that higher dose of Quetiapine resulted in increased incidence of treatment-emergent side effects.

## 4 Discussion

This systematic review is, to the best of our knowledge, the first to investigate the effects of AAPs specifically on psychosocial functioning of patients with a diagnosis of BPD. Results indicated that AAPs may improve global, social, and occupational functioning more than placebo. Additionally, improvements in social functioning were observed over both short and long-term trials and provided early evidence that longer-term AAP treatment may amplify benefits. These observations are important because achieving functional remission would benefit both patients with a diagnosis of BPD and wider society. However, evidence of a clinical meaningful benefit was limited by the small number of included studies, small effect sizes, and poorly reported data by primary studies. Additionally, the concomitant improvement in placebo groups’ functioning was a consistent feature across this review. This is congruous with the surrounding literature (Zanarini et al., 2012a) and may be due to the enhanced psychosocial support associated with trial enrolment (Nenov-Matt et al., 2020) and the ameliorating effects of time itself (Zanarini et al., 2012b).

This review found that AAP treatment improved global functioning more than placebo in trials lasting less than 12-weeks. However, when considering these findings in proportion to the minimum clinically important difference (MCID) in GAF, the clinical relevance of these differences is debatable. Additionally, only two studies were suitable for inclusion in analysis and there was heterogeneity in their reports, thereby limiting the weight of any conclusions. The MCID in GAF has not been defined for BPD populations, so a value of four was adopted following Amri et al. (2014)'s documentations in schizophrenia. Although AAP treatment was associated with baseline-to-endpoint changes exceeding four, the improvement in control groups' functioning meant that the difference between groups' scores failed to reach this cut-off. There is therefore insufficient evidence of a meaningful benefit above that of placebo to justify AAP use for the purpose of improving general functioning. Nonetheless, AAPs did consistently outperform placebo, and if mean weekly changes in GAF were extrapolated it would take approximately 15 weeks for the difference between groups to reach this cut-off. However, it remains unknown whether these trends would continue, and longer-term studies should therefore be conducted. Defining the MCID in populations of patients with a diagnosis of BPD would also increase accuracy of interpretations. BOGENSCHUTZ and GEORGE NURNBERG (2004) was the sole study to individually report statistically significant benefits of AAP relative to placebo, but only at 8-weeks of trial duration and not study endpoint. This implies that Olanzapine is efficacious in improving global functioning, particularly in the context of acute exacerbations of symptoms.

This review found a similar level of evidence for each individual domain of psychosocial functioning investigated. Regarding social functioning, the greatest quality of evidence existed for the ability of AAPs to improve quality of interpersonal relationships. This evidence was of higher quality due to the superior analytical techniques adopted and greater number of included studies. However, the clinical relevance of these observations is challenging to interpret as the MCID for each included scale is unknown. An unexpected observation was that improvements in interpersonal relationship quality increased with longer-term use of Aripiprazole. This may be because longer-term maintenance of AAPs induce more lasting changes in neuromodulation, underpinning improved interpersonal functioning (Morais et al., 2017). Alternatively, early observations may be confounded by the effects of increased support from researchers and clinical staff, which may initially amplify placebo effects (Hansen et al., 1996; Liebke et al., 2017).

Occupational, social-life, and family-life functioning were exclusively assessed using the SDS, and a similar level of evidence existed for all domains. This review found AAP treatment significantly superior to placebo at improving performance within these functional domains by study endpoint. However, the clinical relevance of these differences is less clear-cut and consistent. Regarding occupational functioning, Black et al. (2014) demonstrated that Quetiapine facilitated patients' escape from a highly impaired category (SDS <5), whilst placebo did not. However, these participants had a significantly superior baseline functioning compared to the placebo group, rendering them exquisitely close to this highly impaired cut-off. The meaningfulness of these findings is therefore questionable, particularly considering placebo also enhanced functioning, albeit to a lesser extent. Regarding social-life functioning, Black et al. (2014) failed to demonstrate a clinically meaningful benefit of AAP over placebo. In all functional domains Zanarini et al. (2011)'s results could not be interpreted until the MCID in SDS items is more precisely defined, as participants' baseline functioning exceeded this severely impaired category. The same applied to all measurements of family-life functioning. An added complexity to the interpretation of these results is their purely self-reported nature which is not corroborated by observer-reported data. Patients with a diagnosis of BPD report overly-negative perceptions and memories of themselves which may distort important variations in psychosocial functioning (Lazarus et al., 2014). A combination of self-reported and observer-reported measurement tools is likely superior.

There was some level of heterogeneity between studies. For example, Zanarini et al. (2011) reported greater weekly improvements in GAF in both AAP and placebo groups than Black et al. (2014). One explanation is that poorer baseline functioning in (Zanarini et al., 2011)'s participants may have made individuals more sensitive to improvements. Additionally, Zanarini et al. (2011)'s study was longer (12-weeks versus 8-weeks), administered Olanzapine (versus Quetiapine), and trial protocol differed (for example, fortnightly versus weekly clinic visits). Similarly, Black et al. (2014) reported significant benefits of Quetiapine versus placebo in improving occupational functioning. Their participants had a lower baseline occupational functioning than Zanarini et al. (2011)'s, who reported no significant effect of Olanzapine versus placebo. Lastly, studies' effect size with regards to change in interpersonal relationship quality varied, although the heterogeneity was deemed non-significant. One explanation is varying efficacies of different AAPs on this functional domain, as has been observed with previous reviews investigating other BPD symptoms (Ingenhoven and Duivenvoorden, 2011). If so, the greatest effect was observed for Aripiprazole, followed by Ziprasidone, Olanzapine, and finally Quetiapine. Alternatively, differences in effect size may be due to variations in outcome assessment tool sensitivity, drug doses, or trial protocol or duration. The largest effect was seen in Nickel et al. (2006)'s study, which reported insecurity in social contacts according to the SCL-90-R. The effect size was smaller for the disturbed relationships score of ZAN-BPD, and smaller still for the unstable relations item of CGI-BPD. SCL-90-R is a self-reported tool, relying on an individual's perception of their interpersonal relationships and feelings of discomfort and inadequacy during interactions with others, whilst both CGI-BPD and ZAN-BPD are observer-reported. These internal perceptions may be more sensitive to changes than an external assessor's opinion (Lazarus et al., 2014). A strength of this particular analysis was its incorporation of both these self-reported and observer-reported tools, which together provide a reliable and holistic viewpoint.

The mechanism by which AAPs help patients with a BDP diagnosis in unclear. The APPS selected in the clinical trials form part of what has been called the “second generation mood stabilizers” used for the treatment of bipolar disorder since the mid-1990s (reviewed by Rybakowski, 2023). These drugs were chosen because they exerted a therapeutic effect on manic and/or depressive symptoms during an acute episode and they were efficient for the prevention of recurrence. This is the case for Olanzapine, Quetiapine and Aripiprazole that are used as monotherapy while there are no studies on Ziprasidone monotherapy for the maintenance treatment of bipolar disorder. Interestingly, the reports on online platforms (drugs.com) show that patients feel that the treatments with Olanzapine (68%) and Quetiapine (67%) had clear beneficial effects, while Aripiprazole (56%) seems to produce a slight improvement and too few reports were available with Ziprasidone as it is not so often offered to treat BDP. Most of the beneficial effects reported are related to a down-regulation of dysregulated emotions. Some patients taking Olanzapine describe feeling “calmer and more emotionally stable”, having “no mood swings, no irritability/anger, no depression, no suicidal thoughts, no impulsivity … very little emptiness/boredom.” The stabilization of emotions is probably the main mechanism of action of the AAPs even if during clinical trials the effect seem to be less clear.

Due to the moderate efficacy of the treatment, it is also possible to consider indirect effects. A significant symptom of BDP patients is that they often suffer of insomnia (Vanek et al., 2021). It is becoming clear that sleep plays a fundamental role in emotion regulation and emotional memory processing (Cabrera et al., 2024; Goldstein and Walker, 2014; Rasch and Born, 2013; van der Helm and Walker, 2011). It is therefore possible that some of the AAPs that are known to modify sleep variables, could have also generated in improvement in patients overall social functioning. Polysomnographic studies performed on healthy subjects have shown that Olanzapine, Quetiapine and Ziprasidone administration increased total sleep time and/or sleep. Furthermore, Olanzapine and Ziprasidone augmented slow wave sleep. In patients with schizophrenia, Olanzapine treatment has been shown to induce a significant reduction of sleep latency and an increase of total sleep time and sleep efficiency (reviewed by (Monti et al., 2017)), while quetiapine seemed to disrupt sleep further. However, no study to date has shown the beneficial effects of regulating sleep to improve the emotional well-being include daytime functioning in patients with a BDP diagnostic. Several clinical trials are underway (Reesen et al., 2023; van Trigt et al., 2022) to evaluate the effectiveness of guided cognitive and behavioral therapy for insomnia to reduce symptoms of emotional distress. A better understanding of the role of sleep and its amelioration could have important implications for patients. If the effect quantified in this study were related to improved sleep, then it would open up a new treatment opportunity. Patients could benefit from a combination of pharmacological treatment and/or cognitive behavioural therapy to improve their emotional wellbeing and social functioning that support mental resilience.

It is important to consider adverse effects associated with AAP treatment in proportion to any benefits, in order to maintain patients' health and ensure compliance to treatment regimens. Dropout rates are notoriously high in studies investigating AAP treatment in patients with a diagnosis of BPD (Arntz et al., 2023) due to these side effects and perceived failure of treatment, as noted in the present review. Although significant weight gain was reported overall, findings were heterogenous. Additionally, the present reviews' analyses may be biased towards finding a significant effect on weight gain, as some studies failed to report non-significant results and could therefore not be included in analysis. Heterogeneity may be partially attributable to treatment duration, as weight gain may only emerge after sufficient time has passed. Another explanation is that different AAPs have variable effects on weight. Olanzapine is historically considered to cause the greatest weight gain, due to its actions on serotonin 5-HT2A and 5-HT2C, dopamine D2 and D3, histamine H1, and muscarinic M3 receptors (Dayabandara et al., 2017). Quetiapine generally causes intermediate amounts of weight gain, and Aripiprazole and Ziprasidone cause negligible weight gain even with prolonged exposure (Bak et al., 2014; Dayabandara et al., 2017). The present review's findings are in accordance with these reports, as Olanzapine caused significantly greater weight gain than placebo in two studies, whilst Aripiprazole and Quetiapine did not. Importantly, NICKEL et al. (2007) reported no significant differences in weight gain between those receiving Aripiprazole or placebo after 18-months. Another side effect commonly associated with AAP treatment was sedation, which can itself impair psychosocial functioning. In accordance with the general literature, Olanzapine, Quetiapine and Ziprasidone have moderate sedative effects, much lower than the first generation of antipsychotics, while Aripiprazole induces very little sedation (Eugene et al., 2021). Overall, Olanzapine was most implicated in adverse effects, both in terms of short-term disabling effects and long-term metabolic consequences, whilst Aripiprazole was least often implicated. This is in complete agreement with two previous reviews (Eugene et al., 2021; Stoffers et al., 2010). However, anecdotal patient reports on online platforms, suggest that Olanzapine is the best treatment to stabilise the mood and restore social functioning, whilst the main complaints are related to “emotional state [was] flatlined”, and the associated weight gain made them “self-conscious” (DRUGS.COM, 2021). Knowledge of adverse effects may inform the tailored prescription of AAPs according to patient's medical history and wishes, which may also aid compliance. These adverse effects will become increasingly important to consider if long-term use is recommended.

A strength of this review was its reliance on both self-reported and clinician-reported instruments, which facilitates a comprehensive representation of the effects of AAPs on functional outcomes. Findings were also relatively homogenous as effect direction always favoured the AAP, regardless of outcome or drug. Additionally, several analytical methods were used in conjunction when meta-analysis was not possible. Each analytical method was independently inadequate to answer the research question but contributed unique perspectives and merits to aid interpretation of results. For example, the direction of effect analysis was valuable in presenting the homogeneity of study results, but the sign test output was heavily influenced by the small number of studies. Combining of P-values therefore provided an alternative method of assessing statistical significance, but itself provided no reflection of heterogeneity. This reduced this review's vulnerability to the failings of each statistical method. Additionally, summary statistics were presented, and data was discussed in proportion to the MCID when known, to facilitate interpretation of the findings' clinical relevance.

However, there are limitations to this review derived from the contributing primary studies. Firstly, the failure of several primary studies to report non-significant results may have biased the findings towards the benefits of AAPs. For example, Schulz et al. (2008) failed to report changes in GAF and items of the SDS, instead reporting only items that reached statistical significance. These values could not be included in analysis, although their absence was reported in this review to aid transparency. There was also often limited reporting of standard deviations, standard errors, or P-values which reduced opportunities for robust analysis. This is important as poor-quality or biased analyses may distort the appearance of AAPs’ risk-to-benefit ratio and falsely encourage clinicians to prescribe them. Another limitation was that psychosocial functioning was not the primary outcome of any study. The quality of evidence would be improved if confounding effects and study power were considered in relation to psychosocial functioning a priori (Freemantle, 2001). There was also heterogeneity in primary study methodology with 2/6 allowing concomitant use of other drugs such as benzodiazepines, and 2/6 allowing concomitant psychotherapy. These may confound the effects of AAPs through solitary and interactive effects. Additionally, 5/6 studies were financially supported by the pharmaceutical industry, introducing potential conflicts of interest and biased reporting.

Methodological limitations of this present review include its vulnerability to publication bias, use of the sign test, and method of combining of P-values. As previously discussed, the sign test aids interpretation of the pattern of effect direction, but its power is limited in the context of a small number of studies. Furthermore, the attention to P-values is flawed since these purely represent the likelihood of observing the same result if the null hypothesis was true. They are vulnerable to false positives and provide no information regarding effect size (Karpen, 2017). Another limitation was that patients' baseline functioning differed between studies, which this review did not account for in its analytical methods. Additionally, a comprehensive subgroup analysis of different AAPs' efficacy was not possible due to the limited number of included studies and inability to summarise most data using meta-analysis. Instead, all AAPs were combined in analysis despite the possibility that individual AAPs’ efficacies may vary with regards to each functional domain. This has been reflected in other reviews investigating other symptom domains, and may be because each displays unique receptor binding affinities (Ingenhoven and Duivenvoorden, 2011; Stoffers-Winterling et al., 2022). Similarly, AAP dose varied and was not formally accounted for, which may be another source of heterogeneity. In addition, the studies do not consider the possibility of existing undiagnosed comorbidities. Co-occurring personality disorders in patients with a diagnose of BPD (Grilo et al., 2002; Hastrup et al., 2024) could influence the outcome of AAP treatment. Similarly, responsiveness to the AAP treatment can be affected by an undisclosed substance use, in which case other interventions might be required (Lee et al., 2015). Co-occurrence of autism spectrum disorders (ASD) with BPD could also present a resistance to pharmacological treatment (Barlattani et al., 2023). Lastly, there were a limited number of studies, and certain analyses included a very limited number of AAPs. Evidence of benefit may therefore be missed if the most effective AAP for that functional domain was not included in the analysis.

Areas for further research include conducting longer studies and investigating the impact of study duration. Arguably, these longer-term studies are more relevant than short-term studies. This is because impaired functioning does not pose as an acute risk to patients as other BPD symptoms such as the tendency to self-harm. Instead, the problems associated with difficulty in functioning stem from its tendency to persist and detract from quality-of-life over many years. Determining whether a ceiling effect is reached at a certain timepoint, or whether long-term AAP maintenance is required is also important to define an optimal stopping point of medications to mitigate accumulating adverse effects. It would also be beneficial to determine factors which predict clinical response to better stratify treatment, such as whether symptom severity moderates response. Additionally, research should precisely define the MCID in BPD-specific scales, to accurately determine whether there is a sufficient divergence between AAP and placebo responses to justify their use. Lastly, investigating whether there are interactions with other treatment modalities such as psychotherapy or other pharmacotherapy would be beneficial for real-world applications.

## 5 Conclusion

In conclusion, AAPs convey a small but statistically significant benefit to all domains of patients' psychosocial function, although their clinically meaningful level of added benefit is low or absent. Until superiority is demonstrated in proportion to the MCID in functional assessments specific to populations of patients with a diagnosis of BPD, there is insufficient evidence to justify AAPs' use for the purpose of improving functioning. Additionally, the reported adverse effects are generally more compelling and consistent than the intended effects. This review's findings were also underpowered and undermined by suboptimal methods of analysis. Until further research is conducted it is therefore advisable to utilise treatment methods with a less adverse risk profile, such as psychotherapy, to improve functional capacity (Ripoll et al., 2011). If a clinician chooses to prescribe an AAP for functioning, Aripiprazole may be preferred due to its risk-to-benefit ratio. It has a superior side effect profile and was the most efficacious of the studied drugs for improving quality of interpersonal relationships. Furthermore, it is known to have efficacy in treating other BPD-related symptoms, including identity disturbance and affect regulation difficulties (Stoffers et al., 2010). Regarding future research, it is imperative that primary studies adopt functional measures as primary outcomes, considering the lasting burden of BPD-associated functional impairment. Longer-term primary studies, and subsequent systematic reviews would also be of benefit.

## CRediT authorship contribution statement

**Katie Griffiths:** Writing – original draft, Visualization, Methodology, Formal analysis, Data curation, Conceptualization. **Nadezhda Velichkova:** Writing – review & editing, Visualization. **Lisa Quadt:** Writing – review & editing. **Jimena Berni:** Writing – review & editing, Visualization, Supervision, Resources, Project administration, Funding acquisition, Formal analysis, Data curation, Conceptualization.

## Declaration of competing interest

The authors declare that they have no known competing financial interests or personal relationships that could have appeared to influence the work reported in this paper.

## Appendix

### Search terms

In PubMed the following terms were used to search in titles, abstract, and as medical subject heading terms: “((antipsychotic* or clozapine or olanzapine or risperidone or aripiprazole or quetiapine or ziprasidone or asenapine or paliperidone) and ('Borderline personality disorder' or ‘EUPD’ or 'Emotionally unstable personality disorder'))”

### Fisher's method for combining of P-values

$$X_{2k}^{2}∼-2\sum_{i=1}^{k}\log\left(pi\right)\text{,}$$

### Journal Style and Publication Guidelines

Journal style: SAGE Open.

Publication Guidelines:

“Articles should not exceed 10,000 words (excluding references) and may present original research or literature reviews. The word count (which includes all text including the abstract, manuscript, notes, tables, figures, etc.) should appear on the title page.

Manuscripts should include an abstract of approximately 150 words, and, beneath the abstract, 4–5 keywords. When preparing your abstract, we suggest you describe the purpose of your research, the methods or approaches you used, your results, and your conclusions.

All manuscripts should follow the style guidelines set forth in the sixth edition of the Publication Manual of the American Psychological Association.”

Further details provided here: Manuscript Submission Guidelines: SAGE Open Medicine: SAGE Journals (sagepub.com)
